# Significant Inactivation of SARS-CoV-2 In Vitro by a Green Tea Catechin, a Catechin-Derivative, and Black Tea Galloylated Theaflavins

**DOI:** 10.3390/molecules26123572

**Published:** 2021-06-11

**Authors:** Eriko Ohgitani, Masaharu Shin-Ya, Masaki Ichitani, Makoto Kobayashi, Takanobu Takihara, Masaya Kawamoto, Hitoshi Kinugasa, Osam Mazda

**Affiliations:** 1 Department of Immunology, Kyoto Prefectural University of Medicine, Kamigyo, Kyoto 602-8566, Japan; ohgitani@koto.kpu-m.ac.jp (E.O.); masaharu@koto.kpu-m.ac.jp (M.S.-Y.); hashibirokou8633@ezweb.ne.jp (M.K.); 2 Central Research Institute, ITO EN, Ltd., Makinohara, Shizuoka 421-0516, Japan; m-ichitani@itoen.co.jp (M.I.); m-kobayasi@itoen.co.jp (M.K.); t-takihara@itoen.co.jp (T.T.); h-kinugasa@itoen.co.jp (H.K.)

**Keywords:** novel coronavirus, COVID-19, tea, catechin, theaflavin, polyphenol

## Abstract

Potential effects of tea and its constituents on SARS-CoV-2 infection were assessed in vitro. Infectivity of SARS-CoV-2 was decreased to 1/100 to undetectable levels after a treatment with black tea, green tea, roasted green tea, or oolong tea for 1 min. An addition of (−) epigallocatechin gallate (EGCG) significantly inactivated SARS-CoV-2, while the same concentration of theasinensin A (TSA) and galloylated theaflavins including theaflavin 3,3′-di-*O*-gallate (TFDG) had more remarkable anti-viral activities. EGCG, TSA, and TFDG at 1 mM, 40 µM, and 60 µM, respectively, which are comparable to the concentrations of these compounds in tea beverages, significantly reduced infectivity of the virus, viral RNA replication in cells, and secondary virus production from the cells. EGCG, TSA, and TFDG significantly inhibited interaction between recombinant ACE2 and RBD of S protein. These results suggest potential usefulness of tea in prevention of person-to-person transmission of the novel coronavirus.

## 1. Introduction

The pandemic of novel coronavirus is expanding across the world with nearly 170 million confirmed cases, and global death toll from COVID-19 has exceeded 3.5 million. The SARS-CoV-2 is a Group IV single-stranded positive-sense RNA virus and requires RNA-dependent RNA polymerase for replication [1]. Prophylactic procedures to attenuate the spread of virus infection are needed.

We have explored food ingredients that inactivate SARS-CoV-2, because, if such materials are available, patients as well as healthy people can safely ingest them to eliminate virus from oral cavity and oto- and laryngo-pharynx, and to prevent person-to-person transmission of the virus through droplets.

Tea, including green tea, black tea, and oolong tea, has beneficial effects to human health, such as prevention of neoplasms as well as of cardiovascular, metabolic, neurological, and infectious diseases [2,3,4]. Tea catechins are polyphenolic compounds contained in green tea and contribute to its various biological effects including virus inactivation [5,6,7]. (−) Epigallocatechin gallate (EGCG) is regarded as a representative tea catechin with the highest activity. Tea catechins are quite unstable under certain environments such as alkalic solution and quickly form various multimers by oxidization. Theasinensin A (TSA) is a biologically active black and oolong tea polyphenol, produced by enzymatic oxidation of EGCG. It is one of the major compounds that are formed by incubating EGCG in culture medium [8,9,10]. Theaflavins, typically theaflavin 3,3′-di-*O*-gallate (TFDG), are contained in black tea, because catechins are converted into theaflavins by enzymatic oxidation during the manufacturing of tea leaves into black tea [11,12].

In the present study, we examined whether SARS-CoV-2 can be inactivated by an exposure to tea. We also identified active components that contribute to the anti-viral effect.

## 2. Results

### 2.1. SARS-CoV-2 Was Significantly Inactivated by an Exposure to Tea

**Figure A1:**
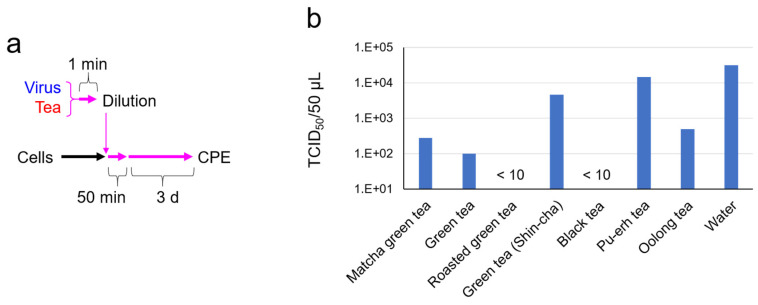
Effects of various tea beverages on infectivity of the novel coronavirus. Powdered matcha green tea, as well as leaves of green tea, roasted green tea (Hojicha), green tea (Shin-Cha, i.e., first picking of a season), black tea, Pu-erh tea, and oolong tea were purchased at a supermarket in Kyoto, and brewed in hot water according to each recipe indicated on the package. After filtrated through a 0.22 μm filter, 500 μL of each sample was mixed with 20 μL of SARS-CoV-2 suspension (5 × 10^5^ TCID_50_/50 μL) and incubated at room temperature for 1 min (*n* = 1). Immediately, each mixture was serially diluted 10-fold into MS, and 50 μL of the samples were added to VeroE6/TMPRSS2 cells that had been cultured in 96-well-plates overnight (*n* = 4 wells per each dilution). Plates were incubated for 50 min with gentle shaking every 10 min. Fifty μL of MS was added to the cells, which were subsequently cultured under standard conditions for 3 days. Cells were stained with Crystal Violet, and TCID_50_ values were calculated by Reed–Muench method as described elsewhere (*n* = 4 wells per each dilution). (**a**) Experimental protocol is shown. Pink arrows represent the presence of tea and virus. (**b**) TCID_50_/50 μL values are shown.

To explore natural substances or food ingredients that inhibit SARS-CoV-2 infection, the virus was treated with various food ingredient materials of botanical origins for 1 min, and the mixtures were subjected to TCID_50_ assays to determine virus titers. As shown in Appendix A Figure A1, the viral titer fell to undetectable levels by the treatment with black tea, while Matcha green tea, roasted green tea (Hojicha), and oolong tea also decreased virus infectivity. Although this was a N-of-1 pilot study in which statistical analysis could not be performed, similar results were obtained from three independent experiments, and we were encouraged to start a series of experiments to examine whether tea inactivates the novel coronavirus.

**Figure 1:**
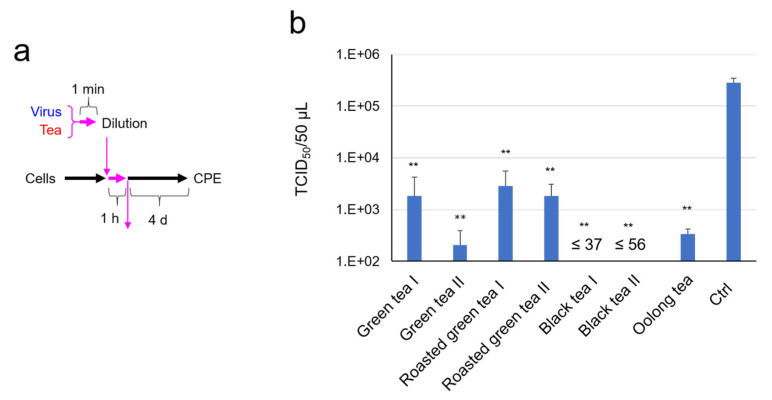
Infectivity of SARS-CoV-2 was significantly reduced by an exposure to tea. Virus was treated with ×1 concentration of the indicated tea/DMEM for 1 min, immediately followed by a 10-fold serial dilution and infection into VeroE6/TMPRSS2 cells to measure TCID_50_ values as described in the Materials and Methods. Scheme of experiments (**a**) and virus titer of each sample (**b**) are shown. Values are means ± S.D. (*n* = 3). ** *p* < 0.01 vs. Control by Tukey’s multiple comparison test.

In the following experiments, freeze-dried powders of each tea were used and osmotic pressure was adjusted by an addition of ×2 DMEM. Virus was pretreated with each tea for 1 min, and the virus/tea/DMEM mixtures were immediately diluted ten-fold. After an addition of the diluted virus/tea/DMEM mixtures to the cells for 1 h, the supernatants were replaced by fresh culture medium (Figure 1a). The TCID_50_ values indicated that black tea strongly inactivated the SARS-CoV-2, followed by green tea, oolong tea, and roasted green tea (Figure 1b).

**Figure 2:**
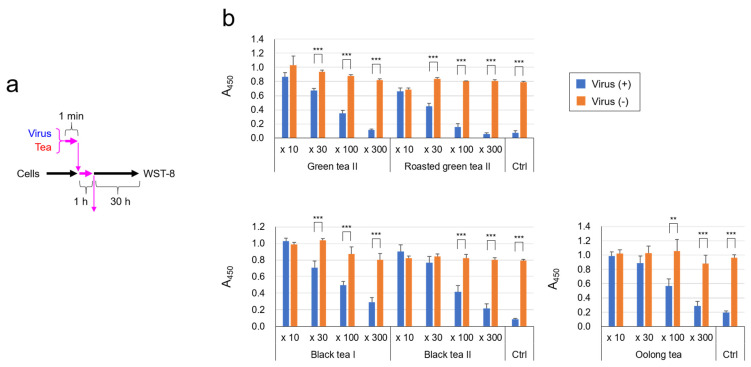
Viability of VeroE6/TMPRSS2 cells after infection with SARS-CoV-2 treated with tea. Virus was treated with each tea at the indicated dilutions for 1 min, and the virus/tea/DMEM mixture was added to VeroE6/TMPRSS2 cells at MOI = 3 for 1 h. After removal of the supernatant, cells were cultured in fresh MS for 30 h, followed by cell viability determination as described in the Materials and Methods. Scheme of experiments (**a**) and A450 value of each sample (**b**) are shown. Values are means ± S.D. (*n* = 3). ** *p* < 0.01 and *** *p* < 0.001 between groups by Student’s *t* test.

Next, the virus was treated with various dilutions of each tea, and after infection cell viability was estimated by WST-based analysis (Figure 2a). Green tea, roasted green tea, and black tea prevented SARS-CoV-2 from damaging VeroE6/TMPRSS2 cells in a dose-dependent fashion (Figure 2b), confirming that tea significantly inactivated the virus.

### 2.2. EGCG, TSA, and Galloylated Theaflavins Significantly Inactivated SARS-CoV-2

**Figure 3:**
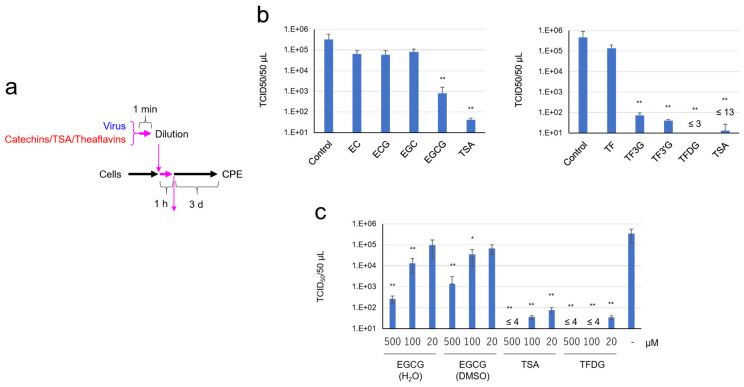
EGCG and galloylated theaflavins significantly decreased SARS-CoV-2 titer. Virus was treated with 500 μM (**b**) or the indicated concentrations (**c**) of tea catechins, a catechin-derivative, and theaflavins for 1 min, immediately followed by a 10-fold serial dilution and infection into VeroE6/TMPRSS2 cells to measure TCID_50_ values as in Figure 1. Scheme of experiments (**a**) and virus titer of each sample (**b**,**c**) are shown. Values are means ± S.D. (*n* = 3). * *p* < 0.05 and ** *p* < 0.01 vs. Control by Tukey’s multiple comparison test.

**Figure A2:**
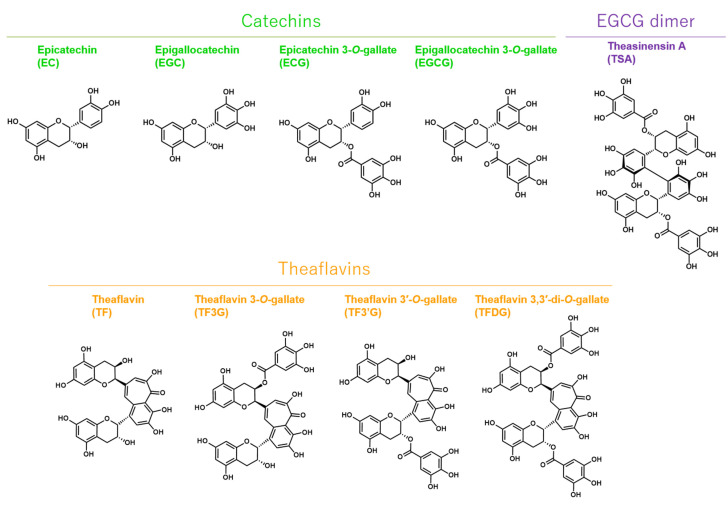
Chemical formulae of the compounds used in the study.

We tried to identify active components that are involved in the anti-virus function. We tested catechins and theaflavins that are well-known functional ingredients in tea (Appendix A Figure A2). First, these compounds were added to the virus suspension at the same concentration (500 µM) to compare the anti-virus activities of these compounds on a molar basis (Figure 3a). As shown in Figure 3b, TCID_50_ assay indicated that SARS-CoV-2 was significantly inactivated by EGCG, but not by other catechins, i.e., (−) epigallocatechin (EC), (−) epicatechin gallate (ECG), and (−) epigallocatechin (EGC). We also prepared theasinensin A (TSA) and examined its effect on SARS-CoV-2. TSA more strongly inactivated the virus than EGCG did. Among theaflavins that we tested, theaflavin 3-*O*-gallate (TF3G), theaflavin 3′-*O*-gallate (TF3′G), and theaflavin 3,3′-di-*O*-gallate (TFDG) strongly inactivated the virus, whereas theaflavin (TF) failed to do so. The anti-virus effects of EGCG, TSA, and TFDG were comparatively analyzed at 20 to 500 µM. As shown in Figure 3c, TSA and TFDG were more effective than the same concentration of EGCG in suppressing SARS-CoV-2, and the treatment with TSA at 500 µM or TFDG at 100 µM caused a significant drop in virus titer to undetectable levels (less than 1/10,000 compared with control).

In the above-mentioned experiments, effects of EGCG, TSA, and TFDG were added at the same doses, to compare the anti-virus activities of these compounds on a molar basis. But these compounds are contained at different concentrations in various tea beverages. As shown in the Appendix A Table A1, the green tea I contained 1.135 µM EGCG, while black tea I contained 40.3 µM TSA and 58.7 µM TFDG. In the next experiments, EGCG, TSA, and TFDG were added to virus at concentrations up to and including 1000, 40 and 60 µM, respectively.

**Figure 4:**
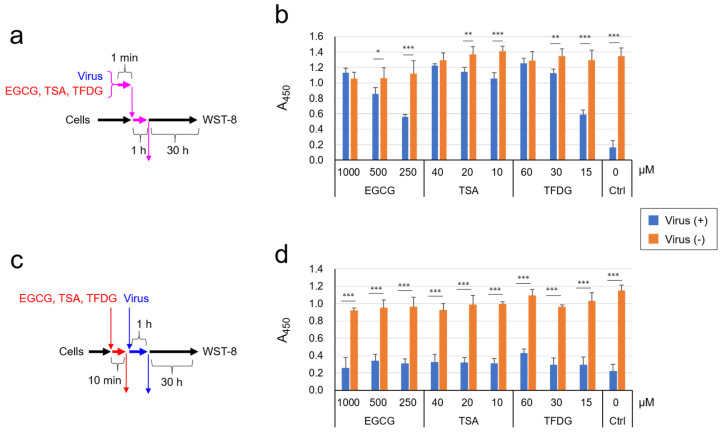
EGCG, TSA, and TFDG inactivated SARS-CoV-2, but did not render cells resistant to intact virus. (**a**,**b**) Virus was treated with each compound at the indicated concentrations for 1 min, and the mixture was added to VeroE6/TMPRSS2 cells at MOI = 3 for 1 h. After removal of the supernatant, cells were cultured in fresh MS for 30 h, followed by cell viability evaluation as described in the Materials and Methods section. (**c**,**d**) Cells were pretreated with the indicated concentrations of the compounds for 10 min, followed by a removal of the supernatant, wash with PBS, and an addition of virus suspension (MOI = 5). One hour later, supernatant was replaced by fresh MS, and cells were culture for 30 h until cell viability was evaluated. Values are means ± S.D. (*n* = 3). * *p* < 0.05, ** *p* < 0.01 and *** *p* < 0.001 between groups by Student’s *t* test.

The virus was treated with EGCG, TSA, and TFDG and subjected to cell viability analysis (Figure 4a). From the results, cell viability was not significantly reduced by infection with the virus that had been treated with 1 mM EGCG, 40 µM TSA, or 60 µM TFDG. Thus, these compounds inactivated SARS-CoV-2 at comparable concentrations to those in tea beverages (Figure 4b).

In other experiments, we preincubated cells with the EGCG, TSA, or TFDG for 10 min, followed by removal of the compounds and infection with untreated virus (Figure 4c). Cell viability analysis showed that the cells pretreated with EGCG, TSA, or TFDG were as highly susceptible to the virus as non-treated cells (Figure 4d). These results were in sharp contrast to the results of the experiments in which untreated cells were infected with EGCG/TSA/TFDG-pretreated virus (Figure 4b), strongly suggesting that the three compounds may directly inactivate virions rather than interact with the cell surface.

**Figure 5:**
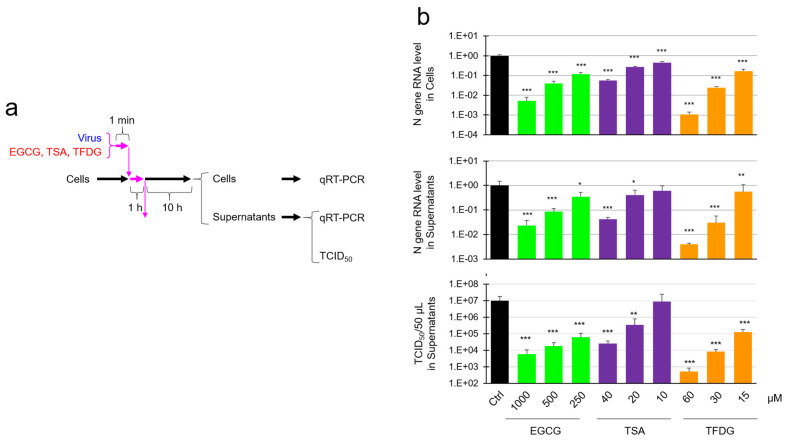
Evaluation of viral RNA in the supernatants and the cells infected with EGCG-, TSA-, or TFDG-treated virus. Virus was treated with the indicated compounds for 1 min, and the mixture was added to VeroE6/TMPRSS2 cells at MOI = 5 for 1 h. After removal of the supernatant, cells were washed with PBS and cultured in fresh MS for 10 h (**a**). RNA was extracted from the supernatants and cells, and real time-RT-PCR was performed as described in the Materials and Methods section. The culture supernatants were also subjected to TCID_50_ assay. Means ± S.D. of relative RNA levels and TCID_50_ values are shown (*n* = 4) (**b**). * *p* < 0.05, ** *p* < 0.01, and *** *p* < 0.001 vs. Control by Tukey’s multiple comparison test.

Real time RT-PCR analysis was performed to evaluate viral RNA in the culture supernatant and the cells that had been infected with the EGCG-, TSA-, or TFDG-treated virus (Figure 5a,b Top panel). As shown in Figure 5b, treatment of virus with 1000 µM EGCG, 40 µM TSA, or 60 µM TFDG resulted in a significant decrease of RNA replication in the cells, and secondary virus released from the cells.

### 2.3. EGCG, TSA, and TFDG Blocked Interaction between ACE2 and RBD of Spike Protein

**Figure 6:**
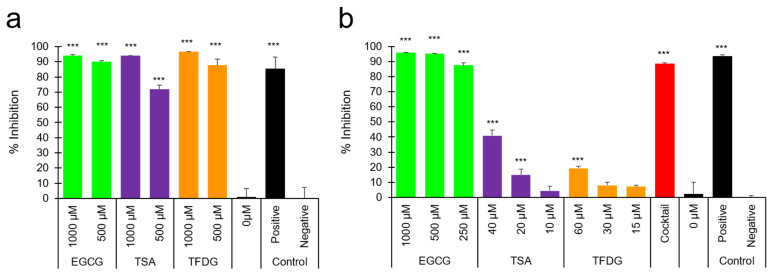
EGCG, TSA, and TFDG interfered with the interaction between ACE2 and RBD. The indicated concentrations of each compound as well as a mixture of 40 µM TSA, 75 µM TF3G, 35 µM TF3′G, and 60 µM TFDG (cocktail) were subjected to a neutralizing assay using a SARS-CoV-2 Surrogate Virus Neutralization Test Kit (GenScript, Piscataway, NJ, USA) as described in the Materials and Methods section. % Inhibition for each sample is shown. Values are means ± S.D. (*n* = 3 (**a**) or 4 (**b**)). *** *p* < 0.001 vs. Negative control by Tukey’s multiple comparison test.

Finally, to figure out the mechanisms of inactivation of SARS-CoV-2 by EGCG, TSA, and TFDG, we estimated influence of these compounds on the interaction between recombinant angiotensin converting enzyme 2 (ACE2) and receptor binding domain (RBD) of the SARS-CoV-2 spike protein. As shown in Figure 6a, these three compounds at 500 or 1000 µM strongly blocked the binding between ACE2 and RBD.

Forty µM TSA and 60 µM TFDG also inhibited the RBD-ACE2 interaction (Figure 6b), but to lesser degrees than 1 mM EGCG. Black tea contained TSA, TF3G, TF3′G, and TFDG that have anti-SARS-CoV-2 activities (Figure 3). Thus, a cocktail composed of 40 µM TSA, 75 µM TF3G, 35 µM TF3′G, and 60 µM TFDG was prepared according to the content of each compound in black tea 1 (Appendix A Table A1). The cocktail remarkably inhibited interaction between RBD and ACE2, strongly suggesting that these compounds in total may strongly suppress viral attachment onto cells when the virus is exposed to back tea.

## 3. Discussion

The present study demonstrated that green tea and black tea, as well as their constituents, EGCG, TSA, and TFDG, significantly inactivated SARS-CoV-2. Concentrations of tea catechins and theaflavins in tea beverages may considerably differ depending on the cultivars of tea plants, procedures and periods of cultivation of the tea plants, levels of enzymatic oxidation during processing of tea leaves, etc. [13]. But green tea and black tea may contain high enough concentrations of EGCG and TSA/TFDG, respectively, for inactivation of SARS-CoV-2. EGCG, TSA, and TFDG at 1 mM, 40 µM and 60 µM, respectively, which are comparable to the concentrations of these compounds in tea beverages, effectively inactivated the virus as demonstrated by cell viability assay, RNA replication, and secondary virus production (Appendix A Table A1, Figure 4b and Figure 5b).

As for the mechanisms of the virus inactivation, our data strongly suggest that EGCG, TSA, and TFDG may interact with virions rather than cells, because pretreatment of virion but not cells with the compounds significantly suppressed infection (Figure 4b,d). Moreover, EGCG, TSA, and TFDG inhibited interaction between recombinant ACE2 and RBD (Figure 6), strongly suggesting that these compounds may interact with RBD of S protein to prevent viral attachment to ACE2 on the cell surface.

Tea catechins are reported to exert various biological effects including anti-oxidation, regulation of lipid metabolism, anti-inflammation, tumor suppression, and suppression of various viruses [3,5,6]. Tea catechins are well known to suppress influenza virus infection by inhibiting attachment of the virions onto the cell surface [6,7]. TSA has similar functions to EGCG, including antioxidant, anti-inflammatory, anti-obesity, anti-cancer, and anti-bacterial functions [14,15]. TFDGs are known to have anti-virus activity in addition to anti-oxidation, anti-obesity, anti-tumor, and anti-inflammatory functions [4,16,17].

To the best of the authors’ knowledge, the etiological correlation between tea consumption and SARS-CoV-2 infection has not been reported.

Clinical significance and application of the present study should be carefully examined in future studies [18]. After oral ingestion of tea, catechins and theaflavins are metabolized by intestinal microbiota and poorly absorbed and transported into blood stream in the gastrointestinal tract [4,19,20,21,22]. Reportedly, the plasma EGCG level reached only 0.6 µM in average after healthy volunteers received an oral administration of 150 mg EGCG twice a day for five days [22]. Plasma concentration of TF peaked at 1.7 nM in healthy volunteers who were given an oral administration of 700 mg mixed TFs [23]. These levels are far below the effective concentrations for inactivation of SARS-CoV-2 (Figure 4b and Figure 5b). Nevertheless, oral ingestion of or gargling with tea, EGCG, TSA, and TFDG may inactivate the SARS-CoV-2 in the oral cavity and pharynx, which may prevent infection by oral entry of the virus. More importantly, ingestion/gargling with tea may suppress person-to-person transmission of the virus, because EGCG/TSA/TFDG may make inactive virions in saliva in infected people [18].

Finally, a lot of people including infants and elderly ordinarily have tea beverages for many years, suggesting that tea and their constituents are safely consumed by anybody. But it should be noted that intake of a large excess amount of catechins could cause adverse events including liver toxicity [13,24].

## 4. Materials and Methods

### 4.1. Virus, Cells, and Culture Medium

The SARS-CoV-2 (previous nomenclature: Japan/AI/I-004/2020; present nomenclature: JPN/TY/WK-521) were kindly provided from Japan National Institute of Infectious Diseases (Tokyo, Japan) and propagated using VeroE6/TMPRSS2 cells. VeroE6/TMPRSS2 cells [25] were obtained from Japanese Collection of Research Biosources Cell Bank, National Institute of Biomedical Innovation (Osaka, Japan) and cultured in Dulbecco’s modified Eagle’s minimum essential medium (DMEM) (Nissui Pharmaceutical Co. Ltd., Tokyo, Japan) supplemented with G418 disulfate (1 mg/mL), penicillin (100 units/mL), streptomycin (100 μg/mL), 5% fetal bovine serum at 37 °C in a 5% CO_2_/95% humidified atmosphere.

### 4.2. Freeze-Dried Powders of Tea Extract

Tea extracts were prepared by soaking 40 g of ground up and homogenized tea leaves in 2000 mL water at 80 °C for 30 min. After centrifugation at 4000 rpm for 15 min, supernatants were collected and filtrated through Toyo No. 2 filter papers, followed by evaporation and freeze-drying.

### 4.3. Catechins, Theaflavins, and a Catechin-Derivative

EC, ECG, EGC, EGCG, TF, TF3G, TF3′G, and TFDG were purchased from FUJIFILM Wako Pure Chemical Corporation (Osaka, Japan). TSA was synthesized from EGCG and purified as described [26]. Briefly, a solution of EGCG (1.0 g, 2.18 mmol) and CuCl_2_ (269 mg, 2.00 mmol) in 30% MeOH (400 mL) was vigorously mixed for 24 h. To the reaction mixture, ascorbic acid (10 g) was added and heated at 85 °C for 15 min. After chilling, the mixture was concentrated to evaporate MeOH and the resulting aqueous solution was applied to a Diaion HP20 column (Mitsubishi Chemical Corp., Tokyo, Japan, 3.0 cm i.d. × 25 cm) with water. After washing the column with water to remove reagents, sample was eluted with 70% MeOH (300 mL), and applied to a preparative high-performance liquid chromatography (HPLC) using a YMC-Actus Triart C18 column (YMC Co., Ltd., Kyoto, Japan, 20 mm i.d. × 250 mm). To elute the column, isocratic elution method was performed using a solvent mixture of water: CH_3_CN = 3:2 at a flow rate of 16 mL/min. LC chromatograms were obtained at UV 280 nm. The fraction of TSA was collected and freeze-dried. The yield and purity of TSA were 251 mg and 96%, respectively.

### 4.4. Measurement of Catechins, Theaflavins, and EGCG Dimers in Tea

HPLC analysis of catechins was performed using an Alliance 2695 Separations Module (Waters, Milford, MA, USA) equipped with 2487 Dual Wavelength Absorbance Detector (Waters). For the separation of compounds, J’sphere ODS-H80 Column (250 mm, 3.0 mm i.d., 4 μm) (YMC, Kyoto, Japan) was used at 40 °C. A total of 10 μL of the sample solutions were analyzed using a gradient elution with a solvent A: ultra-pure water, solvent B: acetonitrile and solvent C: 0.1% phosphoric acid in water. The detection wavelength was set to UV 230 nm. To determine concentrations of theaflavins, LC-MS analysis was performed on ACQUITY UPLC (Waters, Milford, MA, USA) coupled with an ACQUITY TQD (Waters), which were connected by an electrospray ionization (ESI) source. An ACQUITY UPLC BEH Shield RP18 Column (100 mm, 2.1 mm i.d., 1.7 μm) was used at 40 °C for the separation of compounds. An isocratic elution method using [ultrapure water/acetonitrile/formic acid (72.9:27:0.1, *v*/*v*/*v*)] was used to analyze 5 μL of sample solution at a flow rate of 0.5 mL/min. MS was performed with the following parameters: capillary voltage of 2.5 kV, desolvation temperature of 400 °C, and source temperature of 150 °C. Cone voltages were TF 45 V, TF3G 45 V, TF3′G 45 V, and TFDG 55 V, depending on the substance to be analyzed. Single-ion recording was performed in negative mode. To determine concentrations of EGCG dimers, LC-MSMS analysis was performed on ACQUITY UPLC coupled with an ACQUITY TQD, which were connected by an electrospray ionization (ESI) source. An InertSustain C18 Analytical Column (100 mm, 2.1 mm i.d., 2.0 μm) (GLscience, Tokyo, Japan) was used at 40 °C for the separation of compounds. Gradient elution was performed with 0.1% (*v*/*v*) formic acid in water (solvent A) and 0.1% (*v*/*v*) formic acid in acetonitrile (solvent B). Linear elution was performed as follows: 93% A/7% B at 0 min, 93% A/7% B at 1 min, 75% A/25% B at 6.5 min, 74% A/28% B at 9 min, 70% A/30% B at 10 min, 40% A/60% B at 12 min, and 93% A/7% B at 15 min. MS was performed using the following parameters: capillary voltage, 2.7 kV; probe temperature, 600 °C; source temperature, 150 °C; and desolvation gas flow, 600 L/h; cone voltage, 50 V. The mass spectrometer was operated in the negative ESI mode. The peak was detected using an MRM transition for theasinensin A (*m*/*z*. 913.50 > 591.20) in the MS/MS detection.

### 4.5. TCID_50_ Assay for Virus Pretreated with Tea

Freeze-dried powders of green tea, roasted green tea, black tea, and oolong tea was dissolved in sterilized distilled water at 78 °C for 10 min to prepare ×4 concentration of original tea. After chilling at room temperature, each solution was passed through a 0.45 μm filter, and placed into 96-well-plates at 50 μL/well in triplicate. Each sample was mixed with 50 μL of ×2 serum-free DMEM, followed by an addition of 100 μL of SARS-CoV-2 suspension (1.5 × 10^6^ TCID_50_/50 μL) and incubation at room temperature for 1 min. Immediately, the virus/tea/DMEM mixture was serially diluted 10-fold with MS in the 96-well-plates. Chilled on ice, 100 μL of each sample was added to the VeroE6/TMPRSS2 cells that had been seeded into 96-well-plates at 5 × 10^4^/100 μL/well a day before. After culture for 4 days, cells were washed, fixed, and stained with crystal violet solution to estimate CPE as described [27].

### 4.6. Calculation of TCID_50_ Values

TCID_50_ values were calculated by Reed–Muench method as described elsewhere. In some TCID_50_ assays, virus suspension was diluted at 1000-fold before serial dilution as described. Therefore, the detectable limit was higher in these TCID_50_ assays than in others. If one or more triplicate wells of the lowest dilution of a sample did not show CPE, the highest possible average of TCID_50_ value was calculated for the sample.

### 4.7. TCID_50_ Assay for Virus Pretreated with Catechins, a Catechin-Derivative, and Theaflavins

Solutions of EC, ECG, EGC, EGCG, TSA, TF, TF3G, TF3′G, and TFDG were diluted in MS (DMEM supplemented with 0.5% FBS) to concentrations of 2 mM, 400 μM, and 80 μM. The same volume of ×2 DMEM was added to each solution followed by an addition of SARS-CoV-2 suspension (1.5 × 10^6^ TCID_50_/50 μL) and incubation at room temperature for 1 min. Immediately, the virus/compound/DMEM mixture was serially diluted 10-fold with MS, and TCID_50_ assay and calculation of TCID_50_ values were performed as above.

### 4.8. Cell Viability Assay for Virus Pretreated with Tea

Tea was diluted and mixed with the same volume of serum free DMEM as above. Five μL of SARS-CoV-2 suspension (1.5 × 10^6^ TCID_50_/50 μL) or MS was added to 50 μL of each tea/DMEM mixture and incubated at room temperature for 1 min. Immediately, 50 μL of the virus/tea/DMEM mixture was added to the VeroE6/TMPRSS2 cells that had been seeded in 96-well-plates at a density of 5 × 10^4^/100 μL/well a day before (MOI = 3 or 0). One hour later, culture supernatant was replaced by 100 μL of MS, followed by culture for 30 h. To measure cell viability, cells were washed with PBS, and WST-8 solution (Nacalai Tesque, Kyoto, Japan) diluted in phenol red-free DMEM was added to the wells at 50 μL/well. After culture for 45 min, 20 μL of 10% SDS was added to each well and OD at 450 nm was measured. Cell-free wells were regarded as a reference.

### 4.9. Cell Viability Assay for Virus Pretreated with EGCG, TSA, and TFDG

EGCG, TSA, and TFDG was diluted and mixed with the same volume of ×2 DMEM as above. SARS-CoV-2 suspension (1.5 × 10^5^ TCID_50_/50 μL) or MS was added to each mixture and incubated at room temperature for 1 min. Immediately, 50 μL of the virus/compounds/DMEM mixture was added to the VeroE6/TMPRSS2 cells that had been seeded into 96-well-plates at 5 × 10^4^/100 μL/well a day before (MOI = 5 or 0). One hour later, culture supernatant was replaced by fresh 100 μL MS, followed by culture for 27 h. Cell viability was measured as above.

### 4.10. Cell Viability Assay Using Cells Pretreated with EGCG, TSA, and TFDG

Compounds/DMEM mixtures were prepared as above and added to the VeroE6/TMPRSS2 cells that had been seeded into 96-well-plates at 5 × 10^4^/100 μL/well a day before. Ten min later, supernatants were discarded, and wells were washed with PBS, followed by an addition of SARS-CoV-2 suspension (1.5 × 10^5^ TCID_50_/50 μL) to the wells for 1 h (MOI = 5 or 0). After replacement of the culture supernatant by 100 μL of MS, cells were cultured for 27 h, and cell viability was measured as above.

### 4.11. Real Time-RT-PCR

RNA was extracted from culture supernatant using TRI Reagent^®^ LS (Molecular Research Center, Inc., Montgomery Road, Cincinnati, OH, USA) and reverse-transcribed using ReverTra Ace^®^ qPCR RT Master Mix (Toyobo, Shiga, Japan). Quantitative real-time PCR was performed using a Step-One Plus Real-Time PCR system (Applied Biosystems, Foster City, CA, USA) and the following set of primers/probes specific for viral N gene. Forward primer, 5′-AAATTTTGGGGACCAGGAAC-3′; reverse primer, 5′-TGG-CAGCTGTGTAGGTCAAC-3′; and probe, 5′-(FAM) ATGTCGCGCATTGGCATGGA (BHQ)-3′. Ct value for each sample was calculated by StepOne Software (ABI, Warrington, UK). N gene RNA levels in cells were normalized with respect to the 18S rRNA level in each sample. Relative RNA levels (average ± SD) were expressed relative to the value for the control infected with untreated-virus (set to 1.0).

### 4.12. Neutralizing Assay

Neutralizing assay was performed using SARS-CoV-2 Surrogate Virus Neutralization Test Kit (GenScript, Piscataway, NJ, USA) according to the manufacturer’s protocol. Briefly, samples diluted to various concentrations were mixed with a horseradish peroxidase (HRP)-conjugated recombinant RBD fragment at a volume ratio of 1:1 at 37 °C for 30 min. One hundred µL of the mixture was added to the wells pre-coated with human ACE2 protein. After incubation at 37 °C for 15 min, wells were washed and 100 μL of 3,3′,5,5′-tetramethyl-benzidene (TMB) solution was added. Following incubation in the dark at 20–25 °C for 15 min, absorbance at 450 nm was measured.

### 4.13. Statistical Analysis

Statistical significance was analyzed by Tukey’s multiple comparison test (Figure 1, Figure 3, Figure 5 and Figure 6) and Student’s *t* test (Figure 2 and Figure 4). *p* < 0.05 was considered significant.

## 5. Conclusions

An exposure to green tea, roasted green tea, and black tea significantly inactivated SARS-CoV-2 in vitro. The virus was also inactivated by EGCG, theasinensin A (TSA), and galloylated theaflavins including TFDG. The EGCG, TSA and TFDG interacted with Spike protein RBD to prevent interaction between RBD and cellular ACE2. These data suggest the possibility that ingestion of and rinsing with tea may attenuate spread of SARS-CoV-2 [28].

## Data Availability

Not applicable.

## Sample Availability

Samples are not available from the authors.

## Appendix A

**Table A1:** Concentrations (µM) of catechins, theaflavins, and EGCG dimers in the indicated tea used in this study.

	Catechins								Theaflavins				EGCG Dimers	
	GC	EGC	C	EC	EGCG	GCG	ECg	Cg	TF	TF3G	TF3’G	TFDG	TSA	TSD
Green tea I	233	1213	120	440	1135	113	225	108	-	-	-	-	N.D.	N.D.
Green tea II	323	2053	N.D.	613	1173	98	235	95	-	-	-	-	N.D.	N.D.
Roasted green tea I	445	395	153	185	413	183	100	53	-	-	-	-	N.D.	N.D.
Roasted green tea II	370	240	103	95	255	198	73	70	-	-	-	-	N.D.	N.D.
Oolong tea	175	465	58	203	633	65	168	45	N.D.	1.8	2..3	3.6	2.0	7.8
Black tea I	48	73	80	93	240	N.D.	170	50	60.4	76.6	35.8	58.7	40.3	5.1
Black tea II	43	65	53	90	105	N.D.	130	73	51.9	57.1	21.7	29.2	29.8	2.0

N.D. = not detected. - = not determined.
